# Autistic and non-autistic young people’s and caregivers’ perspectives on COVID-19-related schooling changes and their impact on emotional well-being: An opportunity for change?

**DOI:** 10.1177/13623613221140759

**Published:** 2022-12-15

**Authors:** Ann Ozsivadjian, Victoria Milner, Hannah Pickard, Matthew J Hollocks, Sebastian B Gaigg, Emma Colvert, Francesca Happé, Iliana Magiati

**Affiliations:** 1 King’s College London, UK; 2 South London and Maudsley NHS Foundation Trust, UK; 3 City, University of London, UK; 4 The University of Western Australia, Australia

**Keywords:** anxiety, autism spectrum disorders, education services, environmental factors, mental health

## Abstract

Autistic children and young people experience poorer mental health and well-being compared to their non-autistic peers. Navigating the complex social, academic, procedural and sensory aspects of school may be particularly challenging for autistic young people and contribute to poorer mental well-being. The COVID-19 pandemic caused unprecedented school changes and provided a unique opportunity to gather caregiver’s and young people’s perspectives on the impact of school and pandemic-related school changes on the well-being of both autistic and non-autistic young people. Open-text online survey data from 71 caregivers (of *n* = 45 autistic young people) and 30 young people aged 11–18 years (*n* = 18 autistic) gathered across three timepoints between May and December 2020 during the pandemic revealed both benefits and challenges associated with school changes. Insights into possible lessons from the pandemic and recommendations for more flexible, individualised and strengths-based educational practices going forward are discussed.

**Lay abstract:**

Autistic young people experience poorer mental health and well-being compared to their non-autistic peers. Navigating the complex social, academic, procedural and sensory aspects of school may be particularly challenging for autistic young people and contribute to poorer mental well-being. The COVID-19 pandemic caused unprecedented school changes and provided a unique opportunity to gather caregiver’s and young people’s perspectives on the impact of school and pandemic-related school changes on the well-being of both autistic and non-autistic young people. We asked for the views of caregivers and young people aged 11–18 years gathered across three timepoints between May and December 2020. Their responses revealed both benefits and challenges associated with school changes. Insights into possible lessons from the pandemic and recommendations for more flexible, individualised and strengths-based educational practices are discussed.

## Introduction

The high prevalence of co-occurring mental health conditions (e.g. Kent & Simonoff, 2017) and the differential experiences and presentation of mental health difficulties, (e.g. Kerns et al., 2014; Lau et al., 2020; Ozsivadjian et al., 2012) in autistic young people are now well documented. Research papers on prevalence, presentation and interventions for mental health conditions have proliferated, and autism and autistic-led organisations worldwide have raised awareness of both the increased risk and impact of mental health conditions on the lives of autistic people. Cognitive and neurobiological pathways of anxiety in autism have been proposed, including alexithymia (Maisel et al., 2016), cognitive inflexibility (Ozsivadjian et al., 2021) and intolerance of uncertainty (Boulter et al., 2014), furthering our understanding of why and how autistic individuals are much more likely than their non-autistic peers to experience clinically significant anxiety. However, the role of environmental factors, including school, has been less systematically investigated, and therefore models integrating socio-cultural influences and within-child factors are scarce (see Wood & Gadow, 2010 for one example).

A number of qualitative studies have outlined some of the main challenges autistic young people face in school, mainstream or otherwise, via both caregivers’ and young people’s perspectives. These include consistent reports of experiencing social difficulties and negative social experiences with peers, including bullying and vulnerability to exploitation (Sciutto et al., 2012); anxiety and stress in school settings, for example, due to high levels of unpredictability and frequent changes in routine common in school (Humphrey & Lewis, 2008a); and inadequate understanding of autistic young people’s profiles of strengths and needs by school staff (Roberts & Simpson, 2016). Using observational methods, Kasari et al. (2011) found that compared to typically developing peers, autistic young people were more often on the periphery of social networks, reported poorer quality friendships and had fewer reciprocal friendships. Taken together, the impact of the day-to-day challenges faced by many autistic young people in school environments likely has a significant role in the development and/or maintenance of emotional difficulties. Furthermore, these difficulties can often result in a breakdown in educational placement, either due to exclusion (often because of externalising difficulties) or due to internalising problems such as anxiety (Humphrey & Lewis, 2008b) and school refusal (Ochi et al., 2020), which can also lead to long-term feelings of inadequacy, low self-esteem or even traumatic stress reactions (Brede et al., 2017). A recent systematic review by Adams et al. (2020) identified very few studies describing the presentation of anxiety in the classroom, and as the authors highlight, it cannot be assumed that the symptomatology will present in the same way as in children without autism nor that it will be the same as that seen in autistic children within the home setting. Understanding how anxiety presents at school and, crucially, understanding the contribution of school-related factors to higher levels of anxiety are key to improving the experience of young people on the autism spectrum at school.

The COVID-19 pandemic created upheavals to society and the status quo in education that were unprecedented. This provided two potential areas for investigation that particularly pertain to autism and concomitant anxiety – a major change/disruption to existing structures, processes and rules at a societal and local level, and also a complete change in the educational and social milieu. For many, schools were closed and online home-schooling was the only option. For those who were able to attend school (e.g., in the United Kingdom, those with an Education, Health and Care Plan), school was a very different experience, with much smaller class sizes, modified curricula and social distancing measures in place, such as small ‘bubble’ groups of students and one-way systems in corridors. Loss of schooling and potential repercussions for learning has been identified as a major concern for parents of autistic (Daulay 2021; Tokatly Latzer et al., 2021) and non-autistic children (Organisation for Economic Co-operation and Development, 2020). The COVID-19 lockdowns and associated social restrictions therefore resulted in a unique set of circumstances that offered opportunities to investigate the potential influence of school closures, as well as other aspects of changes in school education as a consequence of the social restrictions and/or lockdowns, on autistic young people’s mental health. Alongside other recent studies (Manning et al., 2021; Toseeb & Asbury, 2022), this study therefore aimed to draw on the experiences of autistic children and their families over the COVID pandemic period to identify the most challenging features of the school experience and gain insight into what could be done differently in schools post-pandemic.

Emerging evidence from around the world has demonstrated that the COVID-19 pandemic and related social and school changes have had a detrimental impact on the mental health and well-being of many autistic people and their families (Asbury & Toseeb, 2022; Crane et al., 2021; Manning et al., 2021; Toseeb & Asbury, 2022; White et al., 2021); however, findings across studies are not consistent (Fusar-Poli et al., 2022). For example, caregivers and autistic young people and adults have reported a strong dissatisfaction with the services provided for autistic people during the pandemic, with participants describing a detrimental impact on autistic people’s mental health and well-being (E. Pellicano et al., 2022). However, a subset of participants in E. Pellicano et al.’s (2022) study stated some benefits of the pandemic, such as reduced social demands. Similarly, for a significant minority of autistic children, the removal of certain demands in schools appears to have had positive influences on well-being (Asbury & Toseeb, 2022; Castro-Kemp & Mahmud, 2021; Hurwitz et al., 2022; Tokatly Latzer et al., 2021). Tokatly Latzer et al. (2021) examined the experiences of parents of autistic children and found that although some children experienced worsening of behavioural, social and developmental difficulties, others demonstrated improvement. When interviewing parents of autistic children who attend special education provisions, Hurwitz and colleagues (2022) also found that some autistic children benefitted from, and preferred, the school changes following the pandemic. Asbury and Toseeb (2022) also made similar observations for a significant minority of autistic children in a large survey of parents about their children’s well-being during and after one of the main lockdown periods in the United Kingdom.

Thus, while the COVID pandemic has clearly had detrimental effects on the well-being and mental health of many autistic children and their families, for a substantial number of them the changes in school and wider education provisions appear to have had some positive consequences, which may have important implications for how best to provide adjustments for the diversity of needs across the autism spectrum.

This study therefore aimed to add to the body of literature by qualitatively exploring autistic and non-autistic young people’s experience of school changes and the relationship between these changes and their everyday lives and well-being/mental health, during the COVID-19 pandemic from both caregiver’s and young people’s perspectives. The specific aim was to ascertain what changes to the delivery of education, if any, were seen as advantageous for autistic young people, in terms of both access to education and their emotional well-being, with a view to informing potential recommendations for the educational experiences of autistic young people during and after the pandemic.

## Method

### Participants

Caregivers and their child(ren) (henceforth referred to as ‘young people’), with and without a diagnosis of autism aged 11–18 years, were invited to participate. No exclusion criteria were set with regard to young people’s intellectual functioning or co-occurring conditions. Those with suspected autism and a confirmed autism diagnosis were included.

In total, 71 caregivers completed an online survey: 45 caregivers of autistic young people (including two caregivers currently seeking an autism diagnosis for their child) and 26 caregivers of non-autistic young people (see Table 1 for caregivers’ characteristics). In addition, 30 young people (children of the caregiver participant) participated including 18 autistic and 12 non-autistic young people (see Table 2 for young people’s characteristics). As part of the survey, participants provided details of children’s diagnoses and education settings as well as relevant demographic information.

**Table 1.** Demographic characteristics of caregivers (*n* = 71).

	Autism group (*n* = 45)	Non-autism group (*n* = 26)
Informant		
Mother	39 (87%)	25 (96%)
Father	3 (7%)	1 (4%)
Other	3 (7%)	0
Family country of residence		
England	39 (87%)	25 (96%)
Scotland	1 (2%)	0
Wales	5 (11%)	1 (4%)
Caregiver highest level of education		
GCSEs	4 (9%)	4 (15%)
A level or equivalent	5 (11%)	4 (15%)
Bachelors or equivalent	26 (58%)	12 (44%)
Postgraduate	9 (20%)	4 (12%)
Prefer not to say/missing	1 (2%)	2 (7%)
Child age and age at Dx		
Mean child age (years; *SD*)	15.18 (2.28)	15.06 (1.53)
Mean age at Dx (years; *SD*)	8.4 (3.58)	
Child gender		
Male	24 (53%)	12 (46%)
Female	20 (44%)	14 (54%)
Other	1 (2%)	0
Child ethnicity		
White – British or Irish	35 (78%)	20 (77%)
White – Gypsy or Traveller	0	1 (4%)
White – other	3 (7%)	4 (15%)
Asian	1 (2%)	0
Black – Other	0	1 (4%)
Black – British	1 (2%)	0
Mixed ethnicity	4 (9%)	0
Prefer not to say	1 (2%)	0
Child additional diagnoses		
ADHD	7 (16%)	0
Anxiety	20 (44%)	4 (15%)
Specific learning disability	7 (16%)	0
Other	15 (33%)	2 (7%)
School type		
Mainstream	29 (64%)	24 (92%)
Special needs school	14 (31%)	1 (4%)
Home educated	2 (4%)	1 (4%)
Duration of school attendance in current school		
<6 months	2 (4%)	4 (15%)
6 months–1 year	5 (11%)	3 (12%)
1–2 years	10 (22%)	4 (15%)
2+ years	28 (62%)	15 (58%)
Education health and care plan (EHCP)		
EHCP statement	28 (62%)	2 (8%)
Applying for EHCP	3 (7%)	0
Physical school attendance in first UK lockdown (March–July 2020)		
Yes – no change	3 (7%)	0
Yes – reduced	9 (20%)	1 (4%)
No	33 (73%)	25 (96%)
Schoolwork at home during lockdown period		
Completed schoolwork	23 (55%)	13 (50%)
Partially completed	14 (33%)	8 (31%)
No	2 (5%)	4 (15%)
No – reduced expectation from school	3 (7%)	1 (4%)
Burden of schoolwork on caregiver (1 – no burden, 5 – severe)	*M* = 2.90 (1.52)	*M* = 2.17 (1.11)

Dx: diagnosis; *SD*: standard deviation; ADHD: attention-deficit/hyperactivity disorder; GCSE: general certificate of secondary education.

**Table 2.** Demographic characteristics of autistic and non-autistic youth (*n* = 30).

	Autistic group (*n* = 18)	Non-autistic group (*n* = 12)
Family country of residence		
England	17 (94%)	11 (92%)
Wales	1 (6%)	1 (8%)
Youth age and age of Dx		
Mean child age (years; *SD*)	15.12 (2.03)	15.41 (1.67)
Mean age at Dx (years; *SD*)	7.5 (3.44)	
Youth gender		
Male	14 (78%)	4 (33%)
Female	4 (22%)	8 (67%)
Other	0	0
Youth ethnicity		
White – British or Irish	15 (83%)	9 (75%)
White – other	0	3 (25%)
Black – British	1 (6%)	0
Mixed ethnicity	2 (11%)	0
Youth additional diagnoses		
ADHD	4 (22%)	0
Anxiety	6 (33%)	2 (17%)
SLD	3 (17%)	0
Other	4 (22%)	0
School type		
Mainstream	12 (67%)	11 (92%)
Special needs school	6 (33%)	0
Home educated	0	1 (8%)
Duration of school attendance in current school		
<6 months	0	2 (17%)
6 months–1 year	3 (17%)	2 (17%)
1–2 years	2 (11%)	1 (8%)
2+ years	13 (73%)	7 (58%)
Education health and care plan (EHCP)		
EHCP statement	12 (67%)	0
Physical school attendance during COVID-19		
Yes – no change	1 (6%)	0
Yes – reduced	3 (17%)	0
No	14 (78%)	12 (100%)

Dx: diagnosis; *SD*: standard deviation; SLD: specific learning disability; ADHD: attention-deficit/hyperactivity disorder.

### Materials

The data for the current study were collected in the context of a larger online longitudinal survey during the pandemic, which included demographic questions, questions about children’s educational placement and/or home-schooling circumstances, two standardised questionnaires related to children’s mental health (Revised Children’s Anxiety and Depression Scale; Chorpita et al., 2005; Intolerance of Uncertainty Scale; Carleton et al., 2007) and seven open-ended questions to generate qualitative data regarding caregivers’ and young people’s experience of school changes and their impact on child well-being (see Appendix 1). The qualitative data generated by these open-ended questions are the focus of this article.

These questions specifically asked about aspects of well-being and education, as well as how the COVID-19 pandemic has impacted these. The questions were broad and neutrally worded where possible, and they were informed by anecdotal observations by clinicians in the early days of the lockdown, including some of the study’s authors and the wider literature on the impact of education on mental health of autistic young people (Humphrey & Lewis, 2008a). Thus, their aim was to elicit broad, open-ended descriptions of both the negative and potentially positive consequences of the changing circumstances during the pandemic.

The young people and caregiver versions of the surveys were identical other than wording such as ‘you’ or ‘your child’. Participants completed the survey at three different timepoints throughout the pandemic and the same open-ended questions were asked at each timepoint.

### Procedure

Ethical approval for this study was provided by King’s College London Psychiatry, Nursing and Midwifery Research Ethics Committee (HR-19/20-19466).

Autistic participants and their caregivers were recruited in the United Kingdom via several sources, including the Autistica Discover network, social media, two networks of autistic schools based in London and Sussex, and via an email newsletter for an autism publication called Autism Eye. Non-autistic participants were recruited via social media and mainstream school networks known to the authors. Participants could opt-in to a prize draw to win one of ten £50 gift vouchers.

The study advert explained that we were seeking participation of caregivers of autistic and non-autistic children, as well as autistic and non-autistic young people, in a survey about their experiences of the pandemic. The advert contained a link to an online information sheet with further details about what the survey involved and a consent form. Upon completion of the consent, caregivers were sent an email link to an online survey. If caregiver’s consent was provided, a link to the young people’s survey was also provided via email within the same email as their own survey link. If they followed the survey link, young people were also asked to provide assent and confirm that they understood the study before they could proceed.

**Figure 1.**
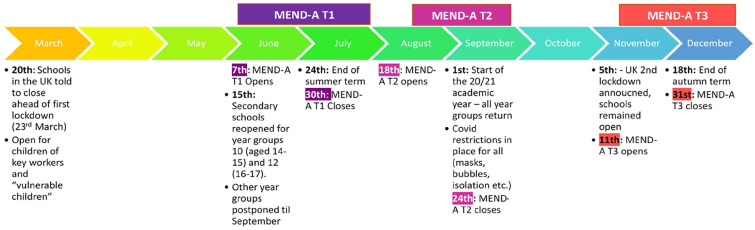
Study procedure and timeline.

Data were collected at three timepoints throughout the pandemic via an online survey hosted on Qualtrics. Timepoints were initially designed to coincide with schools returning to pre-pandemic procedures; however, due to the unpredictable nature of the pandemic, restrictions were still in place. Figure 1 illustrates how these study timeframes mapped onto school-related COVID-19 restrictions and changes.

Timepoint 1 (T1) survey was open for 8 weeks from 7 June 2020 to 30 July 2020. During this survey, participants were asked to consider their (or their child’s) experience before and during the COVID-19 pandemic. Timepoint 2 survey (T2) was open for 5 weeks from 18 August 2020 to 24 September 2020. During T2, participants were asked to consider their (or their child’s) experiences ‘just before the return to school’ prior to the 2020/2021 academic year. Timepoint 3 survey was open for 7 weeks from 11 November 2020 to 31 December 2020. During this survey, participants were asked to consider their (or their child’s) experience ‘6 weeks after returning to school’ following the first term of the 2020/2021 academic year.

### Community involvement

We had informal consultation with autistic individuals and colleagues known to the authors during the conception of the study and survey development; however, there was no formal community involvement in this project.

### Data analysis

Transcripts were analysed by two of the authors, A.O. and V.M., using thematic analysis (Aronson, 1994; Braun & Clarke, 2006). One coder rated by hand and one rated using NVivo software (QSR International, 2020). Data were initially scrutinised to identify information that related to six predefined categories captured within the survey: (1) difficulties faced in school pre-lockdown; (2) benefits of lockdown learning; (3) challenges faced during lockdown learning; (4) general changes to well-being during lockdown; (5) thoughts and attitudes towards return to school after lockdown and (6) caregivers’ and young peoples’ hopes for future changes in education. Adopting an iterative and reflexive process, relevant data from all transcripts were coded to identify themes and subthemes within each predefined category. Themes arising from the three study timepoints were collated and are presented as participants’ overall experiences and perspectives throughout the whole time period of the study. Due to the qualitative nature of the study, we did not seek to quantify differences across timepoints or across groups; however, we were interested to note potential similarities and/ or differences in the reported quality of the experiences between autistic and non-autistic participants in response to the changing circumstances of the pandemic.

Following independent coding, the coding scheme was established and confirmed via discussions between the two coders and applied to the transcripts. Subsequently, one complete transcript was double-coded by both coders to ensure consistency of approach. In addition, two quotes per theme were chosen at random from both the caregiver and young people data and were discussed to determine coherence in interpretation of the data between the two coders. No discrepancies were identified. As we adopted a reflexive analysis approach, it is not appropriate to quantify ‘reliability’ (Braun & Clarke, 2021).

## Results

The qualitative data from both caregivers and young people yielded rich information which is organised in the above-mentioned six categories of questions participants responded to in the survey. A summary of themes derived from both caregivers and young people can be found in Table 3. Quotes from both caregivers and young people are also included to illustrate each theme below. Identifiers are used following each quote to indicate whether the participant was in the autistic (A) or non-autistic group (NA), a caregiver (C) or a young person (Y). Gender and age identifiers are also used; for instance, a quote from a caregiver of a 15-year-old autistic girl would be followed by [AC, girl, 15].

**Table 3.** Summary of thematic analysis domains, themes and subthemes.

Pre-defined category	Themes	Subthemes
1. Difficulties faced in school pre-lockdown	Sensory and environmental difficulties Social challenges and difficulties Lack of appropriate in-school support	
2. Benefits of lockdown learning	Reduced social challenges Increased confidence and motivation Improved access to learning Reduced sensory demands	
3. Challenges faced during lockdown learning	Difficulties engaging with online learning Lack of differentiation between home and school Reduced opportunities for social interaction and other activities Cancellation of exams/lost learning time	
4. General changes to well-being during lockdown	Improvements in well-being Struggling with well-being	Struggling with emotions Struggling with loss of routine and structure Struggling with reduced motivation Struggling with reduced social interaction
5. Thoughts and attitudes towards return to school after lockdown	Apprehension about returning Mixed feelings Positive about returning	
6. Caregivers’ and youths’ hopes for future changes to education	More flexible education for our young people Individualised, strengths-based school learning approaches	

### Category 1: difficulties faced in school pre-lockdown:

We asked caregivers and young people about their experiences of school prior to the pandemic, specifically what aspects they found difficult. Three overarching themes were identified in the data relating to this question.

#### Sensory and environmental barriers

A large proportion of comments by caregivers of autistic young people pertained to difficulty with the school’s environmental/sensory aspects: the number of students, large classrooms and the noisy busy environment: ‘He can be very sensitive to classroom sounds and hubbub which can sometimes be painful and distressing and make it hard to concentrate’ [AC, male, 13]; ‘Classrooms, noise, crowding detentions, teachers yelling at him, hated writing, had difficulty concentrating, switching subjects every hour, hated using public bathrooms, hated eating lunch at school, hated getting the bus, hated wearing a uniform’ [AC, male, 17].

Autistic young people also reflected on the difficulties with the sensory environment at school: ‘I spent the days in an uncomfortable blend of being either uncomfortable over- or under- stimulated with little outlets. I was also perpetually exhausted, no matter how much or how little sleep I got’ [AY, female, 17].

#### Social challenges and difficulties

Social challenges reported by caregivers of autistic young people included ‘Misbehaviour of others’ [AC, other, 16]; ‘My child finds socialising with peers in school difficult’ [AC, male, 13]; ‘She is fundamentally very sociable but completely lost her confidence in mainstream school and became very withdrawn’ [AC, female, 16]. Caregivers of non-autistic young people also reported some social challenges: ‘he is shy and doesn’t handle teasing, so he battles with social interaction, big groups are hard for him to deal with’ [NAC, male, 15]. Very few caregivers of non-autistic young people reported social difficulties beyond ‘only normal teenage friendship issues’ [NAC, male, 16].

Autistic young people also frequently reported experiences of social difficulties: ‘being forced to be in exhausting social situations constantly from which I could not escape, trying to decipher hidden meanings and navigate the complex relationships I didn’t really understand’ [AY, female, 17].

#### Lack of appropriate in-school support

Although caregivers of non-autistic young people reported difficulties with ‘pressure of workload’ [NAC, female, 17], none commented on in-school support. Caregivers of autistic young people described poor access to appropriate support: ‘since he is in the top set for all subjects, teachers forget he needs additional support and clear instructions’ [AC, male, 15].

Inappropriate, or lack of, support was a subtheme prominent within the autistic young people sample, with many participants stating problems such as ‘I didn’t know how to ask the teacher questions [. . .] the school has never given me the amount of support that I need to function well enough in a classroom’ [AY, other, 16].

### Category 2: benefits of lockdown learning:

We then asked more focused questions about the positive impact of the first lockdown on young people’s educational experiences, specifically about the advantages of any changes to education experienced during the lockdown. Four themes were identified in this section.

#### Reduced social challenges

Caregivers of autistic young people commented on a reduction in social stressors. For example, one caregiver said, ‘Some of the hidden challenges faced at school – e.g. social interaction, difficulty with eye contact and other pressures – were removed’ [AC, male, 13]; ‘Not having the daily challenges of attending school, i.e. socialising, coping with classes, coping with the sheer number of students, the noise etc’ [AC, other, 16]. Caregivers of non-autistic young people also commented on the benefits of overall reduced social pressure, although less frequently: ‘She doesn’t have pressure from peers to do well/ exceed and be mocked if she doesn’t’ [NAC, female, 15].

The advantage of being able to learn without social stressors was commented on by autistic young people: ‘I find it a lot easier to focus on my work as I’m not being distracted by idiots in my classes or people talking to me’ [AY, male, 15]. Although these experiences were more prevalent in the autistic young people, non-autistic young people also occasionally reported benefits from the reduced social difficulties: ‘I attended an all-girls school; the social climate was not pleasant and so being away from unpleasant people has been good’ [NAY, female, 17].

#### Increased confidence and motivation

Caregivers of both autistic and non-autistic young people commented on the emergence of new skills, such as self-organisation and increased confidence, for example, in communicating with teachers. For example, ‘My son thrived during lockdown due to the autonomy and self-control he had, leading to improved confidence and removing some of the social challenges faced daily at school and which he quietly copes with’ [AC, male, 13]; ‘he has become more self-motivated and works more independently, he wants to do well and will choose to work on his own, without any nagging from us’ [NAC, male, 15].

#### Improved access to learning

A number of caregivers of autistic young people also commented on the reduced stress impacting on their child’s ability to engage with learning, for example, ‘Much less stress time away from classrooms, time alone, no one yelling at him, no one demanding he pay attention all the time’ [AC, male, 17]. The benefits of 1:1 attention and support from caregivers were also highlighted: ‘we parents are on his side & can make accommodations to reduce the usual daily school stresses’ [AC, male, 15]. Several caregivers of autistic and non-autistic young people commented on the advantages of their children being able to work at their own pace, for example, ‘Choice of activities at his own pace means fewer refusals and less anxiety’ [AC, male, 12]; ‘He considers it to have been far less stress to complete work set when he wanted to, at his own pace without the pressure from teachers’ [NAC, male, 15].

Autistic young people noted that the increased flexibility in managing one’s own timetable and working at one’s own pace was a benefit of lockdown: ‘I also enjoy being able to work independently at my own pace and working in calmer environments at home’ [AY, male, 13]. A subtheme common to both groups of young people was that they were more able to focus at home with fewer distractions: ‘I’ve been able to concentrate better without any distractions’ [NAY, female, 13]. In addition, autistic young people commented that their increased ability to focus was related to better access to coping mechanisms: ‘I have been able to learn without constantly being surrounded by other people and have been able to do things that help me that I usually wouldn’t be able to do, such as stimming or listening to music’ [AY, female, 17].

#### Reduced sensory demands

Finally, caregivers of autistic young people highlighted the advantages of reduced sensory demands: ‘He could use the toilet at home, he could dress in comfortable clothes, he could eat when he was hungry’ [AC, male, 17]; ‘Doing lessons at home without the sensory distractions of school have helped her to do her best and for the teachers to notice her talent / abilities’ [AC, female, 14]. Autistic young people also commented on the sensory advantages, for example, ‘since I’m not in school and not wearing my school clothes, I am in a more relaxed and comfortable atmosphere when doing my schoolwork’ [AY, male, 17].

No comments were made by caregivers of non-autistic young people or the non-autistic young people themselves on the sensory advantages of schooling from home.

### Category 3: challenges faced during lockdown learning

We also asked about the negative impact of the first lockdown on young people’s educational experiences, specifically about the disadvantages of any changes to education experienced during the lockdown. Four themes were identified.

#### Difficulty engaging with online learning

Some caregivers of autistic young people reported their child found it difficult to engage with online lessons: ‘Couldn’t cope with the online lessons – found them hard without staff to help and mostly refused, my son became depressed and angry over this’ [AC, male, 16]; as well as complete disengagement: ‘He’s basically avoiding everything to do with school so we are very concerned about him when he goes back in September’ [AC, male, 17].

Difficulties concentrating/self-motivating at home were also highlighted in both groups of caregivers: ‘She has found home working really tricky, particularly concentrating on a computer screen for long periods’ [AC, female, 14]. ‘Lack of motivation to do schoolwork but, simultaneously, increased anxiety of not completing all the work was set and falling behind’ [AC, male, 15]; ‘Unable to concentrate, no routine’ [NAC, female, 15]. Many comments also pertained to the lack of teacher input and the impact on learning: ‘The lack of conversational style teaching (just notes and PowerPoints to work through) and opportunities to ask the subject experts questions impacted his ability to learn topics well’ [AC, male, 15].

Some young people in both groups commented that their focus and motivation were reduced during lockdown: ‘it was hard to get back into things’ [NAY, female, 16]. Lack of support with schoolwork was also a theme reported by both autistic and non-autistic young people: ‘I am unable to communicate with teachers and classmates in person, which can make navigating through my lessons and tasks harder to do’ [AY, male, 13].

#### Lack of differentiation between home and school

One subtheme that was specific to the caregivers of autistic young people was the lack of differentiation between school and home: ‘My child has had a strong reaction to being home-schooled, he has not liked the fact that school has encroached on home life as he sees his home as somewhere to relax and getaway from school’ [AC, male, 15]; ‘Could not engage with online learning as does not see home as a place to work’ [AC, male, 16]. Caregivers of non-autistic young people did not comment on this.

The autistic young people also commented that a disadvantage to the lockdown changes was that it was difficult to separate school life and home life, being in the same environment all the time: ‘Also it means I no longer feel I am able to get away from schoolwork because home has become school and it feels relentless. It feels like a never-ending cycle I can never get away from’ [AY, male, 15].

#### Reduced opportunities for social interaction and other activities

Caregivers of autistic young people also commented on reduced opportunities for social interaction; for example,

Lack of interaction with people; although in some ways it was very calming for my daughter it has also meant that she is now out of practice. [AC, female, 14]

She has not had any interaction with her school peers since lockdown, this has decreased her confidence in her ability to interact socially. She is not willing to try and has become mute in situations with people she doesn’t know well. [AC, female, 14]

The social disadvantages were also commented on by the caregivers of non-autistic young people: ‘While she is fine with online learning, she is a gregarious person that needs to be with her peers’ [NAC, female, 17]; ‘Needs friends and peer group around’ [NAC, female, 15].

Young people also reported missing social interaction: ‘the lack of proper social interaction is taking its toll’ [NAY, female, 17]; ‘I haven’t been able to meet up with my friends often, so I feel that my wellbeing is partially affected by that. I feel that if I met my friends more often, I would feel happier’ [AY, male, 17].

#### Cancellation of exams and lost learning time

In the group of caregivers of non-autistic young people, a large proportion of the comments pertained to the cancellation of exams as very negative: ‘For children like my daughter who wanted to prove themselves in public exams they will be anxious that their General Certificate of Secondary Education (GCSEs) grades would be considered real’ [NAC, female, 17]; ‘He will not have the experience of taking GCSE exams to prepare him for exams in further education’ [NAC, male, 16].

Both autistic and non-autistic young people were also worried that the lockdown would impact their ability and academic progress: ‘I am scared I will not get the grades I was expecting to get as a result of the lost learning time’ [AY, male, 15].

### Category 4: general changes to well-being during lockdown

To provide a broader context about the impact of the pandemic, we asked both caregivers and young people what effect the changes related to the pandemic in general had on young people’s emotional and mental well-being. Two main themes emerged, improvements in well-being and struggling with well-being. The latter includes four subthemes.

#### Improvements in well-being

Approximately half of the caregivers of autistic young people shared experiences of reduced anxiety – ‘Lockdown was a big relief in my daughter. It took away the stress of having to conform to society’s expectations’ [AC, female, 14].

Very few positive effects of the lockdown were noted by caregivers of non-autistic young people, and of those, many implied that their child had pre-existing anxiety, for example, ‘He is happier being at home than school and as such his anxiety levels have gone down’ and ‘he preferred the peace of doing his studies on his own and as such his anxiety levels have gone down’ [NAC, male, 13]. Others commented on their non-autistic child coping very well with the pandemic and showing incredible maturity: ‘He has coped amazingly well. Very mature outlook and the desire to do the right thing at all times’ [NAC, male, 14].

Emotional benefits were reported primarily by those with an autism diagnosis. One autistic young person reported, ‘My mental health improved massively over the course of the lockdown. As soon as I was out of school, I improved’ [AY, other, 16]. For the autistic young people, the reduced social challenges were a major benefit during lockdown: ‘Since I was not constantly being forced to be in exhausting social situations, trying to decipher hidden meanings . . . or hide “weird” traits such as stimming) I have felt far better in the lockdown due to the lack of social pressure’ [AY, female, 17].

#### Struggling with well-being

##### Struggling with emotions

Both caregivers of autistic and non-autistic young people talked about their children struggling in lockdown emotionally: ‘His already anxious state has become extreme. He vacillates between high anxiety and deep despondency’ [AC, male, 15]; ‘this has not been good for her mental state in lockdown, too much thinking and not enough doing’ [NAC, female, 15].

For caregivers of autistic young people in particular, emotion regulation was a common concern, whereas very few caregivers of non-autistic young people referred to this. As one caregiver noted, ‘More meltdowns, more aggression and anger, higher anxiety and real fear around leaving the house’ [AC, female, 17].

The autistic young people also reported worsened mental health including anxiety, low mood, and worsened self-esteem with more frequency than the non-autistic young people: ‘My mental health has been quite a mess during lockdown. I have been asking myself worried questions, thinking about who I am and what I love’ [AY, male, 17].

##### Struggling with the loss of routine and structure

Far more comments in this subtheme were identified in the autistic caregivers and autistic young people groups: ‘Wants things to be how they were, struggled and is struggling with the changes’ [AC, female, 17]; ‘The sudden changes and restrictions have been really challenging for X, he wants finite answers for unanswerable questions’ [AC, male, 13].

##### Struggling with reduced motivation

Both autistic and non-autistic groups reported loss of motivation: ‘More apathetic about doing anything’ [NAC, female, 17]; ‘He’s struggling with a sense of purposelessness’ [NAC, male, 16]; ‘Become very withdrawn and struggling with motivation’ [AC, female, 18].

##### Struggling with reduced social interactions

In the autistic group, there was more of an emphasis on an exacerbation of existing social difficulties, for example, ‘my child is aware that he is struggling socially, and the lockdown has reinforced this isolation’ [AC, male, 15]; ‘Lack of social connection has been exacerbated by COVID and a reluctance to make connections with people via social media has compounded this’ [AC, male, 15], whereas in the non-autistic caregiver group, comments pertained more to not being able to socialise with existing friends, for example, ‘she missed her friends’ [NAC, female, 15] and ‘lack of interaction led to loneliness’ [NAC, male, 15].

### Category 5: thoughts and attitudes towards returning to school after lockdown

We asked caregivers and young people how they felt about returning to school post-lockdown. Caregivers’ perceptions about their child’s attitude regarding their return to school were mixed for each group. Three themes were identified.

#### Apprehension about returning

Some caregivers of autistic and non-autistic young people reported their child feeling anxious or nervous about the return, for example, ‘[She was] a bit apprehensive [about] new rules, new subjects’ [NAC, female, 15]; ‘very unsure and anxious. Has had no information to help protect yet despite being autistic’ [AC, female, 11].

Autistic young people, more so than non-autistic young people, also shared their concerns about returning:

I am rather apprehensive as I will be attending a new school and haven’t had the best track record at befriending people in the past. I am also rather worried as to how I will balance an increased workload with my friends and my own personal activities such as drawing or researching. [AY, female, 17]

#### Mixed feelings about returning

Many caregivers reported their child felt a range of emotions: ‘Anxious about changes, excited to be doing something different, loss of confidence in own ability but looking forward to getting back to some normality’ [NAC, female, 17]; ‘there was sadness that the long days of autonomy were over but realisation that if she wanted to get GCSEs then school was the best environment to learn’ [AC, female, 14].

Young people in both groups also reported mixed emotions about returning to school, for instance, ‘nervous and scared and excited’ [NAY, female, 17].

#### Positive about returning

In contrast, many caregivers of both autistic and non-autistic young people reported their child felt mostly positive about returning to school: ‘She was thrilled to be back at school amongst people of her own age and excited to start her A level subjects’ [NAC, female, 17]; ‘They were so so happy – like a different person. They couldn’t wait to get back. They were happier, more talkative/communicative . . . a different person’ [AC, female, 18].

Some young people were also excited to return: ‘I feel quite excited coming back to school as it will feel like a unique experience that we do not know much about, and I will enjoy being able to reunite with my school environment’ [AY, male, 13]; ‘I was completely fine returning to school and was looking forward to it’ [NAY, male, 17].

### Category 6: caregivers’ and young people’s hopes for future changes to education

We asked caregivers and young people what their hopes would be for changes to education on their return to school and what lessons could be learnt from this experience. Two themes were identified.

#### More flexible education for our young people

Flexible teaching delivery was noted as a priority by several caregivers of both autistic and non-autistic young people: ‘I would like more part time attendance, with some home learning’ [AC, female, 16]; ‘The ability to have virtual learning and self-management of academic work’ [AC, male, 13]:

I think some of the things her old school are putting in place, such as permanent tutor group, lessons in one room with some exceptions with that same group, one way systems, staggered lunchtimes, particular toilets for their year group, would have made my daughter’s experience of mainstream secondary so much better. The free for all nature of it with 2.4k kids moving around every 45 minutes and often broken toilets was awful for her. [AC, female, 16]

Less homework also came up frequently: ‘less insistence on daily homework’ [AC, male, 13]. Within the caregivers of the non-autistic young people group, a few also shared a hope for the continuation of some online learning, including online learning options as a method to catch up if falling behind.

The autistic young people, but not the non-autistic young people, also expressed their hope for more flexible timetables, including the option for virtual lessons and/or assignments:

I hope the option to do remote learning will still be a thing when COVID-19 lockdown restrictions are eased, or even remote learning of sorts when the virus is entirely contained. I also hope there will be a way for students to complete work in their own time and schedules, as well as a way for students to learn in a manner where there is less going on in the surroundings. [AY, male, 13]

Several autistic young people also commented that they would prefer some COVID-related school-based restrictions, such as staggered start times and one-way corridors, to remain in place even after the pandemic ends: ‘In terms of in-person schooling, we begin later at staggered times to other years so there are less people when moving around the school’ [AY, female, 17].

#### Individualised, strengths-based school learning approaches

There was also a hope for greater understanding of each child’s strengths and needs, especially for caregivers of autistic young people. For example, caregivers commented on how this experience revealed that teachers need more training in how to approach and support autistic young people, with a focus on strengths as opposed to difficulties, to bolster confidence, self-esteem and well-being. The following two quotes were particularly poignant:

That my child will have more autonomy and that the school will allow a more child-led, individual approach. [AC, male, 13]

Strengths should be focused on, rather than what a child cannot do, to bolster confidence, self-esteem and wellbeing. Some of the positives brought by lock-down should be incorporated into new norms at school and in society generally, as the world reboots and to make life easier for children with differences. [AC, male, 13]

## Discussion

In this study, we aimed to evaluate autistic and non-autistic young people’s educational experiences and the impact on their well-being during the COVID-19 lockdown in the United Kingdom. In this unprecedented period of rapid change, we had an opportunity to understand the experiences of young people and their caregivers during a time of radical changes to education delivery, including, by necessity, flexible education practices, home-based and online approaches to learning. We asked participants to reflect upon and contrast these with their pre-COVID experiences of their formal school-based education.

In terms of general emotional well-being, caregivers of both autistic and non-autistic young people described a range of negative effects. Specific to the well-being of the autistic group was the impact of the loss of routine and support services. Common across both groups, and shared by caregivers and young people alike, was the reduced social contact, resulting in isolation or loneliness, and some also reported increases in anxiety and depression. In line with E. Pellicano et al. (2022), we found that autistic young people also felt socially isolated and missed having social contact during lockdowns, despite preconceptions that autistic people might be less socially motivated than non-autistic people (Chevallier et al., 2012). Therefore, when considering why social stressors lead to anxiety in autism, one important aspect to consider is that both the quality and amount of social interaction/connections are likely to lead to difficulties with anxiety and mood, rather than a simple linear relationship between social experiences, stress and social anxiety.

However, one striking difference between responses of autistic and non-autistic young people or their caregivers was the reporting of several perceived benefits of the lockdown circumstances and changes for autistic young people, in particular with regard to their educational experiences. Fewer caregivers of non-autistic young people or young people themselves reported benefits. These included reduced social pressure, reduced sensory overload, not having to conform and not having to hide one’s autism-related characteristics, such as stimming behaviours. Overall, a reduction in stress and anxiety was reported by several caregivers and autistic young people alike. This finding aligns with those of recent studies, which have also found that a significant minority of autistic individuals (and their caregivers) experienced improvements in well-being and mental health during school closures (Asbury & Toseeb, 2022). Among the possible reasons for this observation are that experiences of bullying (Sciutto et al., 2012), feelings of being misunderstood (Roberts & Simpson, 2016), or high levels of anxiety linked to sensory processing differences and/or high intolerance of uncertainty, can make aspects of the school environment particularly distressing for autistic young people (Humphrey & Lewis, 2008a). It is likely that the specific reasons for the improvements in well-being and mental health reported by many autistic young people during school lockdown are varied. The fact that such experiences were reported by a significant minority of autistic young people, however, suggests that there is room for more successful adjustments to their education provisions.

The main aim of this article was to delineate which pre-covid educational practices were the most problematic for autistic young people and which, if any, of the novel educational practices, could be retained to improve their educational experiences. To this end, we specifically asked people what the advantages and disadvantages were of educational practices during the lockdown. Advantages such as working at one’s own pace, reduced sensory demands, reduced social demands resulting in reduced anxiety overall, which in turn improved the learning experience were reported by many caregivers and autistic young people. These advantages, unsurprisingly, corresponded to the difficulties described by caregivers of autistic young people and the young people themselves during conventional education pre-lockdown, which in turn mirrored the challenges identified in existing research, namely sensory challenges, social challenges, processing differences, and feeling misunderstood or autism-related needs not being met by teaching staff (National Autistic Society, 2017).

Disadvantages of online schooling from home during lockdown, particularly for the autistic young people and their caregivers, included difficulty focusing during online learning and reduced social interaction. For autistic young people only, the lack of differentiation between home and school was reported to be problematic. The overlap between advantages and disadvantages for some participants highlights the need for individualised approaches to education delivery, rather than a ‘one size fits all’ approach (see also Asbury & Toseeb, 2022); for example, for some young people, online learning reduces classroom distractions, but for others, it resulted in difficulty maintaining motivation and attention while also increasing the feelings of loneliness and social isolation.

The hopes outlined by caregivers and autistic young people indicated that many practices or changes made during the pandemic were welcome and there was a wish for these to continue flexibly. These align well with the recommendations made by a number of educators from the Pan London Autism Schools Network (PLASN) group (Crane et al., 2021) and include facilitating holistic approaches to support, ensuring clear and regular communication with families, providing effective support for home learning and promoting collaborative ways of working. As the authors noted, many of these initiatives align with best practice principles in autism education more generally (e.g. Guldberg 2019). In their article ‘The Current Illusion of Educational Inclusion’, L. Pellicano et al. (2018) highlight a lack of consensus regarding how best to achieve inclusion, and reasons for failure to achieve true inclusion, and potential solutions, including cultural change and better awareness of the social and learning challenges of autistic students at all levels. This period of change may well have provided us with some of the practical solutions to achieve these aims.

### Strengths and limitations of this study

Qualitative research generally aims to be as non-directive as possible; however, as we wished to examine specific issues relating to young people’s educational experiences during the pandemic and their impact on learning and well-being, the nature of some of our questions was directive. We attempted to balance these by asking either neutrally worded questions or asking about positives and negatives of each issue being examined.

Survey methodology has the advantage of being far-reaching as it can be advertised widely and reduces the need for travel or arranging a time with a researcher. At times of high levels of social restrictions, it was also the only methodology available to carry out research exploring these issues. However, the amount of information participants provide in response to open-ended questions within online surveys varies, and while some of our participants provided a good deal of information, others did not complete some or all of the qualitative questions. In addition, the autistic group was roughly twice the size of the non-autistic sample, thus providing a richer source of data to identify themes from, and as with all volunteer-based research, participants in our online survey were self-selecting in response to study adverts. As a consequence, the participants may not be representative of the wider population, or of circumstances outside the UK context, and we cannot be sure that our results would generalise to other autistic and non-autistic young people or their caregivers. Nevertheless, the findings converge with other qualitative and quantitative work examining factors that contribute to anxiety and difficulties with aspects of school in autism (e.g., Asbury & Toseeb, 2022; Brede et al., 2017; Toseeb & Asbury, 2022) and underline the importance of considering what further adjustments could be made to education provisions to effectively support the diverse needs of autistic children. Importantly, the researchers coding the data were not autistic, and therefore, it is possible that interpretation might not fully reflect the autistic person’s experiences and meaning.

## Summary and conclusion

Our study attempted to integrate caregivers’ and young people’s reflections and experiences about the challenges of everyday school experiences pre-COVID, with their novel insights from the radical changes to education delivery that were implemented throughout the pandemic. Most importantly, we sought to highlight the relevance and importance of educational experiences to young people’s overall mental well-being; a link which has been largely neglected so far in the literature. Our participants gave clear descriptions of the social, organisational and sensory challenges of everyday school experiences and how some of the lockdown changes to education suited the needs of some autistic individuals. In some ways, this was an enforced naturalistic experiment introducing novel ways of learning and schooling, providing a unique opportunity to effect real change. COVID-related educational changes also provide, an opportunity to question the previous status quo and recognise that traditional schooling was not working for many young autistic people who experience social exclusion, educational exclusion and mental health crises because they are overwhelmed with the demands of conventional schooling.

Parents and young people expressed a desire for the following:

- Continued flexible learning;
- A more individual, strengths-based and young people-led approach to learning;
- Greater understanding of individual needs and learning profiles.

To conclude, we thought that one quote from a caregiver of an autistic young person captured particularly well the essence of what we hope readers will take away from this study:

Many adults with autism found that their anxiety began as children because they were not diagnosed or misunderstood, and I think this is a really good time to make change. I believe that as a society, as a world, we must not go back to certain habits and this involves our educational provision for children with special needs. [AC, male, 13].

## Appendix 1

Open-ended questions included in the survey:

1. What aspects of their usual (pre-COVID-19) school placement did your child find difficult?
2. Regarding their educational experience, what have been the ADVANTAGES/BENEFITS of the COVID-19 lockdown for your child?
3. Regarding their educational experience, what have been the disadvantages/challenges of the lockdown for your child?
4. Are there any changes to your child’s educational experience that have occurred during the COVID-19 lockdown that you hope will remain in place when the COVID-19 lockdown is eased?
5. Are there any changes to your child’s educational experiences that you hope will be made after the COVID-19 lockdown is eased?
6. Aside from school changes, is there anything else significantly impacting your child’s well-being during the COVID-19 lockdown (e.g. Changes in medication regime, significant life events, lack of access to professionals or other formal/ informal supports, other?)?
7. Is there anything else that you would like to tell us about your child’s mental health and educational experience during the COVID-19 lockdown?
